# Development and validation of a machine learning-derived dual-indicator tool for identification of hepatitis B virus-related pre-acute-on-chronic liver failure

**DOI:** 10.3389/fcimb.2026.1865757

**Published:** 2026-07-27

**Authors:** Tianyi Zhang, Shaoli You, Jinjin Luo, Jun Ling, Sa Lv, Yiwen Xu, Shaojie Xin, Xiaohe Xiao, Jun Li, Bing Zhu

**Affiliations:** 1 Chinese PLA General Hospital, Beijing, China; 2 Senior Department of Hepatology, Fifth Medical Center of Chinese PLA General Hospital, Beijing, China; 3 State Key Laboratory for Diagnosis and Treatment of Infectious Diseases, National Clinical Research Center for Infectious Diseases, National Medical Center for Infectious Diseases, The First Affiliated Hospital, Zhejiang University School of Medicine, Hangzhou, China

**Keywords:** ACLF (acute-on-chronic liver failure), early prognosis, HBV related pre-acute on chronic liver failure, machine learning, user-friendly tool

## Abstract

**Background & aims:**

Patients who do not meet the criteria for Acute-on-Chronic Liver Failure (ACLF) at admission still face a high risk of disease progression and mortality. This study aimed to develop and validate a machine learning-derived clinical tool for the early identification of HBV-pre-ACLF.

**Methods:**

We analyzed 1,682 patients experiencing acute deterioration of HBV-related chronic liver disease but without ACLF. By random forest and SHAP analysis, we identified key predictors of ACLF onset within a 7-day window. We then validated the diagnostic thresholds in a test cohort of 260 patients.

**Results:**

Total bilirubin (TBIL) and INR were identified as the most critical predictors of ACLF progression. We established optimal diagnostic thresholds at TBIL ≥131.5 μmol/L and INR ≥1.35. Two sequential criteria were developed: pre-ACLF-O (meeting either threshold) for highly sensitive screening, and pre-ACLF-A (meeting both thresholds) for risk confirmation. In the derivation cohort, pre-ACLF-O achieved 98.3% sensitivity, while pre-ACLF-A predicted a 33.09% positive predictive value. These results were successfully validated in the test cohort, where pre-ACLF-O maintained 100% sensitivity and pre-ACLF-A demonstrated a 37.04% positive predictive value.

**Conclusions:**

This dual-indicator tool easily and effectively identifies HBV-pre-ACLF. Using these criteria sequentially provides a practical strategy for early risk stratification, allowing clinicians to initiate timely interventions for high-risk patients.

## 1 Introduction

The definition and underlying etiologies of acute-on-chronic liver failure (ACLF) vary significantly between Eastern and Western populations. In Western countries, ACLF is predominantly driven by alcohol-induced liver injury and chronic hepatitis C, frequently presenting as a syndrome of multiple organ failure precipitated by extrahepatic infections (Bajaj et al., 2022) (EASL clinical practice guidelines on acute-on-chronic liver failure, 2023). Conversely, in the East, chronic hepatitis B virus (HBV) infection is the primary underlying liver disease, with HBV reactivation serving as the principal trigger for acute hepatic insults (Sarin et al., 2019). Initially, HBV-ACLF may manifest as single organ (liver) failure in its early stages, resembling ACLF as defined in Western countries when infection occurs due to compromised immunity. The current consensus, EASL-ACLF, describes ACLF as a syndrome with a high 28-day mortality rate (≥15%) (Moreau et al., 2013), primarily induced by primary hepatitis in the context of chronic liver disease.

Recognizing these distinct pathophysiological pathways, the Chinese Group on the Study of Severe Hepatitis B (COSSH) developed the COSSH-ACLF criteria. By adjusting diagnostic indices such as total bilirubin (TBIL) and international normalized ratio (INR) specifically for HBV-ACLF, these criteria successfully standardized 28-day mortality assessments to align with Western benchmarks (Wu et al., 2018). Subsequently, validation analyses conducted by the COSSH working group confirmed that the China CLIF framework (originally designated as the COSSH-ACLF criteria) demonstrated consistent prognostic accuracy for both all-cause and population-specific manifestations of ACLF (Luo et al., 2025).

**Figure 1:**
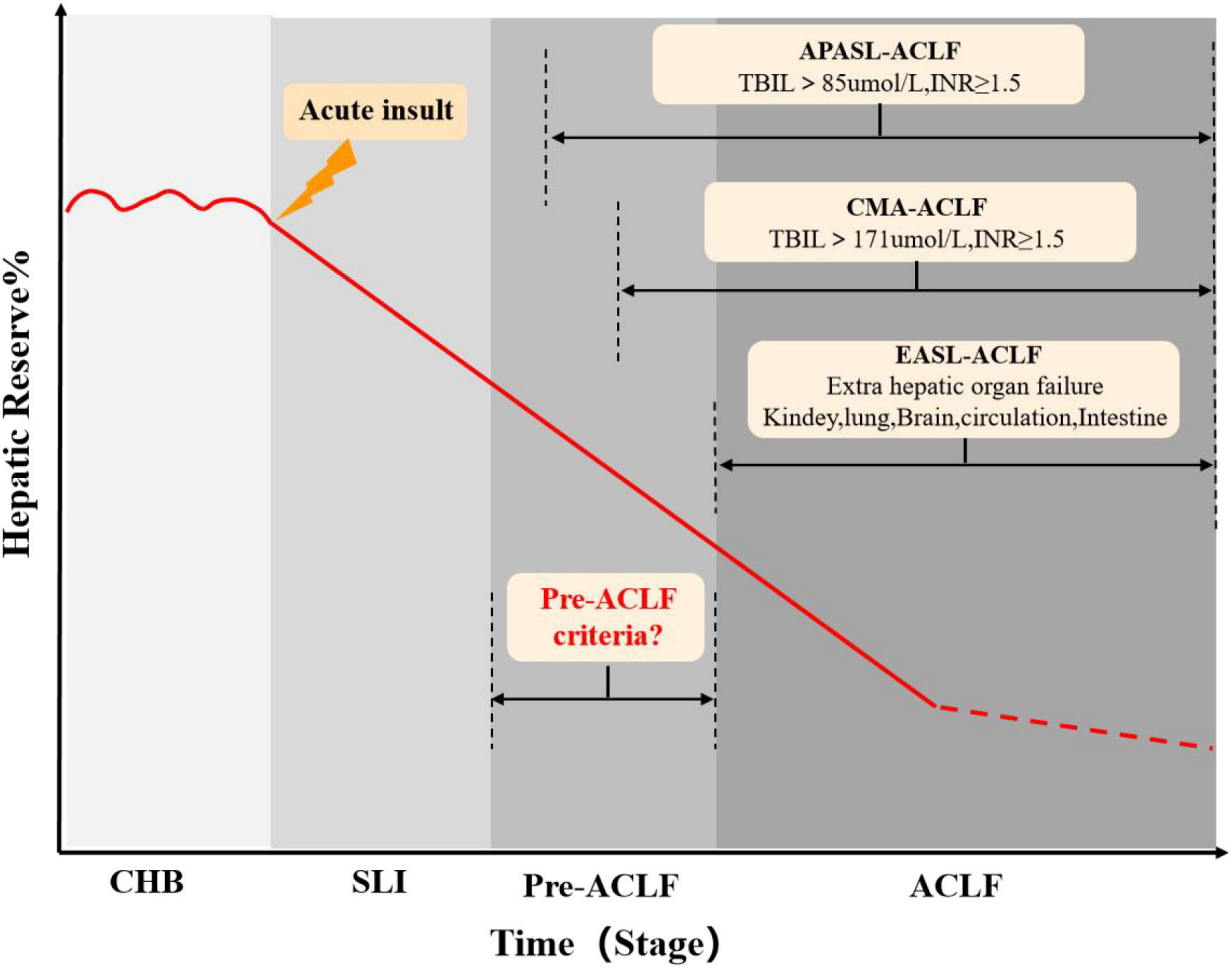
Schematic comparison of Pre-ACLF with current ACLF standards.

Eastern ACLF criteria, such as those by the Chinese Medical Association (CMA) and the Asian Pacific Association for the Study of the Liver (APASL), appear less severe compared to EASL-CLIF criteria when using the conversion index of COSSH. According to the Asian Pacific Association for the Study of the Liver (APASL) criteria, ACLF is indicated by TBIL≥85.5 μmol/L, INR≥1.5, while the Chinese Medical Association (CMA) criteria require TBil≥171μmol/L, INR≥1.5 for an ACLF diagnosis (Shi et al., 2015). Patients with chronic Hepatitis B may experience acute deterioration in liver function when exposed to certain triggers, initially presenting as Subclinical Liver Injury (SLI; TBIL ≥ 85.5 μmol/L in patients with chronic hepatitis B), progressing to pre-ACLF, and eventually ACLF. Despite advances in diagnostic frameworks, currently there is no universally accepted diagnostic standard for HBV-pre-ACLF. Because established HBV-ACLF carries a notoriously high short-term mortality rate, early identification and timely clinical intervention during the pre-ACLF window are essential to halt disease progression. The COSSH research team established a unified understanding of the concept of HBV-Pre-ACLF (**Figure 1**). The COSSH team previously developed a complex prognostic model for ACLF onset based on this open cohort; however, it did not yield a clear diagnostic criterion for HBV-pre-ACLF.

To address this critical knowledge gap, this study applied a machine learning-based framework to analyze clinical data from patients experiencing HBV-induced acute liver function deterioration. Our primary objective was to refine the clinical characteristics of the pre-ACLF stage and develop a novel, convenient dual-diagnostic tool to improve the early identification and management of high-risk patients.

## 2 Patients and methods

### 2.1 Study design and population

This study utilized a two-phase methodological approach to develop and validate a diagnostic tool for pre-ACLF. Patients hospitalized for a minimum of three days due to acute deterioration of HBV-related chronic liver disease were initially screened and subsequently enrolled from the prospective open cohort of the COSSH study (Wu et al., 2018). All patients received comprehensive treatment, which included antiviral agents for those with HBV DNA positivity, management of ascites, hepatic encephalopathy (HE), and bacterial infections, as well as renal replacement therapy for hepatorenal syndrome. The derivation cohort comprised patients admitted between January 2017 and December 2019. An independent prospective test group, used for validation, included patients admitted between January 2020 and November 2021. Acute deterioration of HBV-related chronic liver disease was defined by two primary clinical presentations: (1) patients with a known history of chronic hepatitis B exhibiting severe liver injury, defined as TBIL ≥ 85.5 μmol/L; and (2) patients with diagnosed cirrhosis presenting with acute complications, such as ascites, upper gastrointestinal bleeding, hepatic encephalopathy, bacterial infections, or significantly elevated jaundice (TBIL ≥ 85.5 μmol/L).

By comparing clinical predictive factors between patients who progressed to ACLF within seven days of enrollment and those who did not, we sought to identify independent risk factors. We selected risk indicators with strong correlations, alongside traditional clinical indicators, to predict pre-ACLF, establishing cutoff values based on the receiver operating characteristic (ROC) curve. Subsequently, we trained a random forest model to predict the onset of pre-ACLF. Given the significance of risk factors associated with prognosis, we carefully selected the primary indicators to formulate a novel user-friendly prognostic diagnostic criterion. Clinical and follow-up data were collected via electronic case report forms.

### 2.2 Definitions and clinical endpoints

#### Definition of ACLF criteria

##### CMA-ACLF

According to the 2018 edition of the CMA guideline (Guideline for diagnosis and treatment of liver failure, 2019).

##### APASL-ACLF

As per the APASL-AARC criteria, Acute-on-Chronic Liver Failure (ACLF) is characterized by a sudden liver injury leading to jaundice (total bilirubin ≥5 mg/dL or 85 μmol/L) and coagulation abnormalities (INR ≥1.5 or Plasma Thromboplastin Antecedent ≤40%), accompanied by clinical ascites and/or encephalopathy within a 4-week period in a patient with known or unknown chronic liver disease/cirrhosis. ACLF is linked to a significant 28-day mortality rate (Sarin et al., 2019).

##### EASL-CLIF

Per EASL criteria, the diagnostic criteria for ACLF is established through the presence of three main characteristics: Acute decompensation (AD), organ failure (assessed by the Chronic Liver Failure Consortium Sequential Organ Failure Assessment score), and a high 28-day mortality rate (defined by a threshold of 15%) (Moreau et al., 2013).

Pre-ACLF was defined by two primary characteristics: chronic hepatitis patients experiencing acute liver function deterioration upon exposure to a specific factor, leading to Subclinical Liver Injury (SLI), and a rapid progression to liver failure within 7 days (with a defined threshold of 30%). Patients with severe liver injury (total bilirubin [TBIL] ≥ 85.5 µmol/L and international normalized ratio [INR] ≥ 1.5) due to chronic hepatitis B (CHB) were classified as COSSH-SLI according to the COSSH study.

#### Clinical endpoints

While major international criteria (such as EASL-CLIF, APASL-ACLF, and CMA-ACLF) define established ACLF based on varying thresholds of jaundice, coagulopathy, and organ failure, this study focused on the critical window immediately preceding ACLF onset. The primary clinical endpoint was the progression to ACLF within a 7-day observational timeframe. This 7-day period was strategically selected to balance early risk stratification with clinical feasibility, providing clinicians sufficient time to evaluate treatment efficacy before irreversible progression occurs.

### 2.3 Ethics approval

The study protocol was approved by the local ethics committee, the Clinical Research Ethics Committee of the First Affiliated Hospital, Zhejiang University School of Medicine (no. 2017-51). Informed consent was obtained from the patients or their first relatives if they could not provide informed consent themselves before enrolment.

### 2.4 Statistical and machine learning analysis

IBM SPSS Statistics 25 was used for the analysis of baseline indicators. The chi-squared test was used to compare categorical variables. Normally distributed data are expressed as mean ± standard deviation and were compared using an independent-samples (Student's) t-test. Non-normally distributed data were analyzed using the Mann–Whitney U-test and are expressed as an interquartile range. Receiver operating characteristic (ROC) curves were plotted using MedCalc 15.2.2 to find cut-off values. GraphPad Prism 9.0.1 was used for graphing. Differences were considered significant at p < 0.05. A random forest algorithm was trained to predict the 7-day onset of ACLF using the caret package in R (4.0.1). Using the caret, random Forest (RF) and pROC packages, the data were divided into training and validation sets in a 7:3 ratio. The models were trained using the train function in the caret package. To address class imbalance, random oversampling was applied only to the training data within each cross-validation fold.

Model performance was rigorously assessed via 10-fold cross-validation, utilizing confusion matrices to evaluate classification accuracy. Thereafter, the trained model was used to form predictions using the validation data. Receiver operating characteristic (ROC) curves were generated by R. To ensure the clinical interpretability of the machine learning model, feature selection and variable importance were evaluated using the SHapley Additive exPlanations (SHAP) algorithm. In addition to statistically assessing indicators with high correlation coefficients, commonly used and internationally recognized indices were selected for differentiation. Finally, the cut-off value was subsequently applied to identify optimal clinical thresholds for the most predictive features.

## 3 Results

### 3.1 Baseline clinical characteristics

From an initial screening of 2,601 patients with acute exacerbations of HBV-related chronic liver disease, 1,682 patients did not meet the criteria for ACLF at the time of admission and were included in the derivation cohort. Within the 7-day observation window, 118 patients rapidly progressed to ACLF, while 1,559 did not. Additionally, five patients underwent liver transplantation following admission (**Supplementary Figure 1**).

Baseline comparisons revealed distinct clinical profiles. The clinical characteristics of patients who developed ACLF, as defined by the EASL-ACLF criteria, and those who did not develop ACLF upon admission are detailed in **Table 1**. Non-progression group presented poorer specific laboratory indicators upon admission than developed ACLF group.

**Table 1:** Baseline characteristics at admission of patients who did or did not develop ACLF by day 7.

Variable	Variable classification	Developed ACLF (N=118)	non-Developed ACLF (N=1559)	p.value
Age(yr)		45 (35, 60)	50 (41, 58)	0.063
Antivirals		25 (21.2%)	543 (34.8%)	0.004
Cirrhosis		63 (53.4%)	1141 (73.2%)	<0.001
Hepatic encephalopathy grade	S0	114 (96.7%)	1500 (96.2%)	0.643
	S1	1 (0.8%)	33 (2.1%)	
	S2	2 (1.7%)	17 (1.1%)	
	S3	1 (0.8%)	9 (0.6%)	
	S4	0	0	
Ascites	0	67 (56.8%)	739 (47.4%)	0.033
	1	26 (22.0%)	302 (19.4%)	
	2	21 (17.8%)	467 (30.0%)	
	3	4 (3.4%)	51 (3.3%)	
Infection		24 (20.3%)	390 (25.0%)	0.305
TP (g/L)		58.10 (54.825, 62.975)	59.90 (54.7, 64.3)	0.211
Alb(g/L)		31.33 ± 4.44	31.31 ± 5.78	0.961
Glo(g/L)		26.75 (22.725, 31.375)	27.50 (23.4, 32.3)	0.316
ALT(U/L)		431.00 (134.25, 809.5)	48.00 (23, 241)	<0.001
AST(U/L)		305.00 (126.500, 529.250)	63.00 (32, 158.5)	<0.001
ALP(U/L)		138.50 (115, 169)	108.00 (75, 141)	<0.001
TBA(μmol/L)		194.50 (155.150, 282.650)	71.00 (17.000, 165.900)	<0.001
TBIL(μmol/L)		185.25 (155.350, 203.950)	62.00 (21.200, 145.150)	<0.001
DBIL(μmol/L)		135.25 (109.750, 171.850)	34.70 (10.000, 116.000)	<0.001
IBIL(μmol/L)		48.15 (35.175, 59.225)	18.90 (10.000, 33.500)	<0.001
r-GT(U/L)		104.00 (73.000, 145.750)	61.00 (27.000, 129.000)	<0.001
CRE(μmol/L)		65.00 (57.000, 75.000)	68.00 (59.000, 79.000)	0.010
Serum urea (mmol/L)		3.89 (2.807, 5.683)	4.83 (3.660, 6.700)	<0.001
TG (mmol/L)		1.27 (0.973, 1.845)	1.01 (0.680, 1.840)	0.001
TC (mmol/L)		2.46 (2.092, 2.908)	2.78 (2.280, 3.397)	<0.001
HDL-C(mmol/L)		0.21 (0.133, 0.340)	0.53 (0.230, 0.870)	<0.001
LDL-C(mmol/L)		0.86 (0.383, 1.343)	1.24 (0.780, 1.730)	<0.001
GLU (mmol/L)		4.32 (3.433, 5.647)	4.66 (4.050, 5.730)	0.003
K(mmol/L)		4.11 (3.737, 4.475)	3.96 (3.660, 4.270)	0.002
Na(mmol/L)		139.00 (137.000, 140.750)	139.00 (137.000, 142.000)	0.071
CRP (mg/L)		10.10 (6.495, 16.900)	6.80 (3.300, 13.300)	<0.001
Serum ammonia(μmol/L)		51.50 (33.750, 68.000)	39.00 (24.000, 59.000)	0.005
HBsAg		2399.01 (427.618, 9671.590)	827.39 (95.210, 2488.700)	<0.001
HBV-DNA level		1075000.00 (14925.000, 20625000.000)	5170.00 (0.000, 806000.000)	<0.001
WBC (10^9 /L)		6.05 (4.525, 7.700)	4.50 (3.100, 6.100)	<0.001
Neutrophil (10^9 /L)		3.70 (2.900, 5.250)	2.70 (1.800, 3.900)	<0.001
Lymphocyte (10^9 /L)		1.23 (0.930, 1.550)	1.00 (0.600, 1.490)	<0.001
HGB(g/L)		131.50 (121.000, 145.750)	113.00 (89.000, 133.000)	<0.001
HCT(L/L)		38.55 (34.000, 42.950)	33.30 (26.700, 38.800)	<0.001
PLT (10^9 /L)		109.50 (79.250, 150.750)	85.00 (52.000, 143.000)	0.002
INR		1.82 (1.412, 2.345)	1.35 (1.210, 1.540)	<0.001
FIB (g/L)		1.42 (1.175, 1.770)	1.57 (1.225, 2.040)	0.005
PT(s)		20.40 (16.425, 26.500)	15.50 (13.900, 17.600)	<0.001
D-dimer (ug/L)		1581.50 (726.250, 3518.750)	1235.00 (451.000, 3468.000)	0.051
AFP (ng/mL)		48.45 (13.600, 163.225)	8.25 (2.400, 86.975)	<0.001
Ferritin(ng/mL)		2380.30 (1077.400, 4007.575)	420.90 (86.000, 1431.850)	<0.001

Categoric variables are expressed as n (%); continuous variables are expressed as either means ± SD or median [interquartile range]. P values are comparisons between ACLF and non-ACLF (Student t test, Mann–Whitney U test, chi-squared test, or Fisher exact test). ACLF, acute-on-chronic liver failure; TP, total protein; Alb, albumin; Glo, globulin; ALT, alanine aminotransferase; AST, aspartate aminotransferase; ALP, alkaline phosphatase; TBA, total bile acid; TBIL, total bilirubin; DBIL,Direct Bilirubin; IBIL, Indirect Bilirubin; γ-GT, γ-glutamyl transferase; CRE, creatinine; TG, triglyceride; TC, total cholesterol; HDL-C, High-Density Lipoprotein Cholesterol; LDL-C, Low-Density Lipoprotein Cholesterol; GLU, glucose; CRP, C-reactive protein; HBV, hepatitis B virus; WBC, white blood cell count; HGB, hemoglobin; HCT, Hematocrit; PLT, Platelet Count; INR, international normalized ratio; FIB, Fibrinogen; PT, Prothrombin Time; AFP, Alpha-Fetoprotein.

### 3.2 Machine learning feature selection

**Figure 2:**
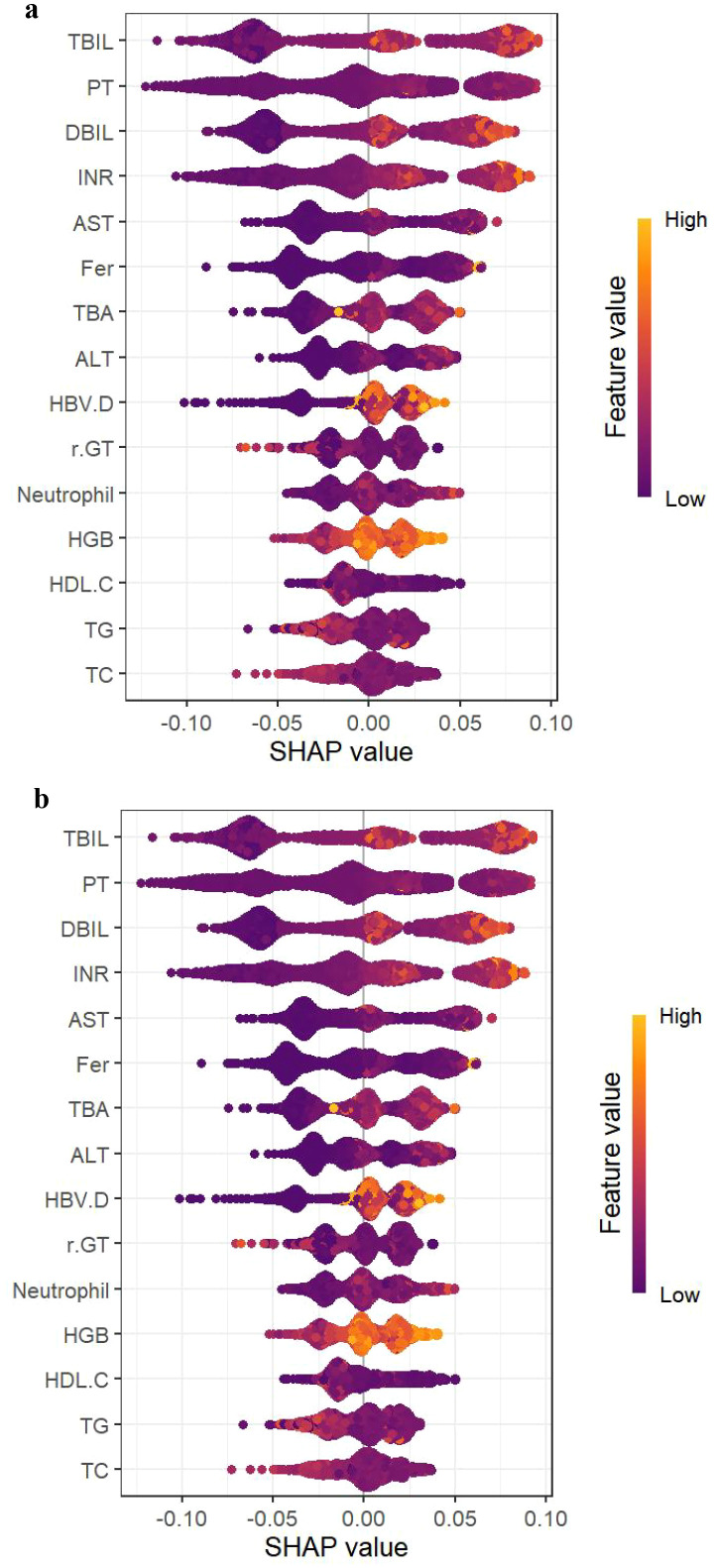
**(a)** Feature selection of different variables to predict COSSH-SLI patients progressed into ACLF within 7 days using SHAP in derivation group. **(b)** Feature selection of different variables to predict COSSH-SLI patients progressed into ACLF within 7 days using SHAP in test group.

The random forest model was applied to baseline clinical indices to establish the predictive model of ACLF progression. The ROC curve from the random forest analysis is illustrated in **Supplementary Figures 2a, b**, and the model’s sensitivity and specificity are detailed in **Supplementary Table 1**. SHAP analysis identified TBIL, INR, ferritin, direct bilirubin (DBIL), and prothrombin time (PT) as the most critical features driving the model’s predictions. The significance of confirmed variables in derivation and test group is illustrated in **Figures 2a, b**. Because TBIL and DBIL are clinically redundant and PT reference ranges vary regionally, TBIL and INR were selected as the core indicators for developing a practical diagnostic tool because they are both standardized, universally recognized markers.

### 3.3 Development of the dual-indicator diagnostic tool

We emphasize TBIL and INR due to their reliability and accuracy in diagnosing ACLF. ROC curve analyses for the selected predictors established optimal predictive cut-off values at 131.5 μmol/L for TBIL and 1.35 for INR (**Supplementary Figures 3a, 3b**). To balance the need for high sensitivity (for screening) and high specificity (for targeted intervention), patients were stratified using a dual-criteria framework.

Patients presenting with either TBIL ≥ 131.5 μmol/L or INR ≥ 1.35 were classified under the “pre-ACLF-O” (Either/Or) criteria. Patients presenting with concurrent TBIL ≥ 131.5 μmol/L and INR ≥ 1.35 were classified under the “pre-ACLF-A” (And) criteria.

In the derivation cohort, the pre-ACLF-O criteria identified 1,164 high-risk patients, successfully capturing 116 of the 118 individuals who progressed to ACLF within 7 days, achieving a sensitivity of 98.3%. Concurrently, the more stringent pre-ACLF-A criteria identified 284 patients, 94 of whom rapidly progressed to ACLF, yielding a highly positive predictive value of 33.09%.

### 3.4 Validation of the dual-indicator criteria in an independent cohort

The dual-indicator tool was subsequently evaluated in an independent test cohort comprising 260 patients, 32 of whom (12.31%) developed ACLF within 7 days. When applied to this test set, the pre-ACLF-O criteria identified 212 at-risk patients, successfully capturing all 32 subsequent progressions, thereby achieving 100% sensitivity. The pre-ACLF-A criteria identified 81 patients, of which 30 developed ACLF, demonstrating a consistent and specific incidence rate of 37.04%.

## 4 Discussion

Acute-on-chronic liver failure is a rapidly progressive syndrome with high mortality, where liver transplantation often remains the only definitive treatment (EASL clinical practice guidelines on acute-on-chronic liver failure, 2023) (Kulkarni et al., 2023). Research indicates that patients can develop ACLF within 7 days, even if they do not initially present with ACLF at admission. Moreover, the 28-day mortality rate for those initially diagnosed without ACLF is significantly high. Clinical studies have shown a marked decrease in the incidence of disease progression with early therapeutic measures (Angeli et al., 2015) (Erstad, 2023) (Sole et al., 2018). It is advised that these patients receive standardized ACLF treatment promptly following diagnosis. Because clinical outcomes improve significantly with early and intensive therapeutic intervention, identifying patients during the transitional “pre-ACLF” phase is critical (Xia et al., 2013) (Chen et al., 2019) (Li et al., 2018). This study successfully developed and validated a machine learning-derived, dual-indicator diagnostic framework to standardize the early identification of HBV-related pre-ACLF.

It is critical to emphasize that the clinical management of pre-ACLF must be etiology-specific, as diverging underlying mechanisms dictate distinct prognoses and therapeutic strategies (Zhu et al., 2014) (Avila et al., 2020) (You et al., 2013). For example, alcohol-related and HCV-related ACLF typically present as infection-precipitated multi-organ failure rather than isolated hepatic dysfunction. In contrast, HBV-ACLF characteristically initiates as isolated liver failure, only subsequently progressing to multi-organ failure driven by secondary insults such as infection (EASL clinical practice guidelines on acute-on-chronic liver failure, 2023) (Sarin et al., 2019). This distinct pathophysiological trajectory explains why liver-specific indices, namely TBIL and INR demonstrate such robust predictive performance specifically within the context of HBV-ACLF.

Furthermore, staging the evolution of HBV-ACLF facilitates highly customized clinical management. During the pre-ACLF window, aggressive antiviral therapy and hepatic support are paramount to halt progression. Conversely, once full ACLF develops, the clinical focus must urgently shift toward managing systemic complications, including infection, hemodynamic shock, hemorrhage, and multi-organ dysfunction. Ultimately, formalizing the pre-ACLF concept establishes a vital window for early, disease-modifying intervention. This phase-appropriate approach not only improves treatment success rates but also optimizes the allocation of medical resources, thereby reducing overall healthcare costs.

Traditional scoring systems, such as the Model for End-Stage Liver Disease (MELD), MELD-Na scores, and Child-Pugh scores, have limited utility for prognostic assessment in this setting. Recent landmark initiatives, such as the PREDICT study and the Ch-CLIF.C study, have sought to formally conceptualize the “pre-ACLF” state. The PREDICT study identified patients with pre-ACLF as those who developed ACLF within 90 days, characterized by high-grade systemic inflammation at enrollment and a substantial 90-day mortality rate of 67%. While this model demonstrating high specificity (95%) but low sensitivity (38%) (Trebicka et al., 2020) (Tang et al., 2023) (Wang et al., 2022). In related research, including the acute decompensation of cirrhosis study and the Ch-CLIF.C study conducted in China, hospitalized patients with acute decompensation (AD) who progressed to ACLF within 28 days were categorized as pre-ACLF. Patients exhibiting one or fewer organ dysfunctions had a significantly reduced risk of developing ACLF within 28 days and could be reliably excluded, with a pre-ACLF misclassification rate of less than 5%. The Chinese pre-ACLF classification proposed by Lanjuan Li's group similarly indicated a poor prognosis for these patients. In previous investigations (Luo et al., 2023), the COSSH Group developed a mathematical model to predict the onset of HBV-ACLF, facilitating early and accurate prognostication. The COSSH team also evaluated the broader applicability of the China-CLIF framework across diverse aetiologies and varying severity levels of ACLF (Luo et al., 2025). It is crucial to acknowledge that the definitions of pre-ACLF employed in these studies were derived from clinical observations, and there is currently no universally accepted standard for this classification. Although these observational frameworks are clinically valuable, they currently lack a universally adopted, easily calculable bedside standard. By leveraging random forest modeling and SHAP interpretability, our study systematically established a random forest model and isolated TBIL and INR as the primary drivers of ACLF progression.

We derived precise thresholds (TBIL ≥ 131.5 μmol/L and INR ≥ 1.35) and integrated them into a practical dual-criteria system. The pre-ACLF-O criteria (meeting *either* threshold) serve as a highly sensitive screening mechanism. In our derivation and test cohorts, this approach achieved sensitivities of 98.3% and 100%, respectively, minimizing false negatives and ensuring deteriorating patients are not overlooked. Conversely, the pre-ACLF-A criteria (meeting *both* thresholds) function as a highly specific, confirmatory diagnostic standard. With approximately one-third of patients meeting the pre-ACLF-A criteria progressing to full ACLF within a week (33.09% in the derivation cohort, 37.04% in the test cohort), this pre-ACLF-A criteria allows clinicians to confidently deploy high-intensity, resource-heavy interventions—such as artificial liver support—while avoiding unnecessary financial strain on lower-risk patients.

The clinical utility of this dual-indicator tool lies in its sequential application. The pre-ACLF-O criteria (meeting either threshold) serve as an aggressive, highly sensitive screening mechanism, minimizing false negatives and ensuring that no potentially deteriorating patient is overlooked. Conversely, the pre-ACLF-A criteria (meeting both thresholds) act as a specific, confirmatory diagnostic standard. Because nearly one-third of patients meeting the pre-ACLF-A criteria progress to full ACLF within a week, this tool empowers clinicians to confidently deploy high-cost, high-intensity interventions—such as artificial liver support systems, while avoiding unnecessary financial strain on lower-risk patients.

In this study, we propose an innovative framework that integrates the pre-ACLF-O (meeting either TBIL or INR criteria) and pre-ACLF-A (meeting both TBIL and INR criteria) definitions, aiming to synergize the diagnostic advantages of both. We recommend a two-step, hierarchical clinical management strategy. First, pre-ACLF-O should be utilized as an initial screening tool to identify patients at high risk for disease progression; its high sensitivity facilitates early detection and significantly minimizes the risk of missed diagnoses. Subsequently, if a patient’s condition progresses to meet the pre-ACLF-A criteria, clinicians can confidently initiate timely, comprehensive interventions and intensive monitoring. The high specificity of the pre-ACLF-A criteria substantially reduces false positives and misdiagnoses, ensuring that aggressive treatment is accurately targeted.

By sequentially combining a highly sensitive screening threshold with a highly specific diagnostic confirmation, we have constructed a stratified management pathway for patients with acute exacerbation of HBV-related chronic liver disease. This complementary approach not only optimizes overall diagnostic efficiency but is also highly practical; because TBIL and INR are routinely measured laboratory parameters, these indicators are simple, cost-effective, and easily adopted in everyday clinical practice.

## 5 Conclusions

The prognosis and progression of ACLF vary significantly depending on the underlying etiology. Focusing specifically on HBV-related disease, we propose a novel, dual-indicator framework utilizing baseline TBIL (≥ 131.5 μmol/L) and INR (≥ 1.35) to accurately identify patients at high risk of rapid progression to ACLF. The pre-ACLF-O criteria (meeting either threshold) provide a highly sensitive early warning system to promptly capture potential deteriorations. Subsequently, the pre-ACLF-A criteria (meeting both thresholds) offer a definitive, highly specific diagnostic standard, successfully predicting rapid progression in over one-third of identified cases. Together, this sequential diagnostic tool provides a simple, accessible, and evidence-based foundation for the early identification, risk stratification, and targeted management of pre-ACLF in clinical practice.

## Data Availability

The data generated and/or analyzed during the current study are available with the corresponding authors upon reasonable request.
