# Supplementary material for: Development and validation of a machine learning-derived dual-indicator tool for identification of hepatitis B virus-related pre-acute-on-chronic liver failure
Source: Front Cell Infect Microbiol. 2026 Jul 27;16:1865757. doi: 10.3389/fcimb.2026.1865757 (PMC13454397; doi:10.3389/fcimb.2026.1865757)
Supplement: Supplementary file 1 [file Supplementaryfile1.doc]

**Supplementary Table 1.** The sensitivity, specificity of random forest model to predict COSSH-SLI patients progressed into ACLF within 7 days.

| **Group** | **Sensitivity (%)** | **Specificity (%)** | **95%CI** |
| --- | --- | --- | --- |
| the derivation group | 100 | 100 | 0.9957, 1 |
| The test set | 65.79 | 84.38 | 0.6876, 0.7778 |

**Supplementary Figure 1.** Screening, enrollment and classification of patients. ACLF, acute-on-chronic liver failure; LT, liver transplantation. TB, total bilirubin; INR, international normalized ratio. *LT, liver transplantation within 7 days of admission before the onset of ACLF.

**Supplementary Figure 2a.** ROC curves of random forest model to predict COSSH-SLI patients progressed into ACLF within 7 days in derivation group.

**Supplementary Figure 2b.** ROC curves of random forest model to predict COSSH-SLI patients progressed into ACLF within 7 days in test set.

**Supplementary Figure 3a/3b.** ROC curves of different variables and models to predict COSSH-SLI patients progressed into ACLF within 7 days.
